# MicroRNA-34a/EGFR axis plays pivotal roles in lung tumorigenesis

**DOI:** 10.1038/oncsis.2017.50

**Published:** 2017-08-21

**Authors:** Y-L Li, X-M Liu, C-Y Zhang, J-B Zhou, Y Shao, C Liang, H-M Wang, Z-Y Hua, S-D Lu, Z-L Ma

**Affiliations:** 1 Lab for Noncoding RNA & Cancer, School of Life Sciences, Shanghai University, Shanghai, China; 2 Experimental Center for Life Science, Shanghai University, Shanghai, China; 3 Shanghai Sixth People’s Hospital Affiliated to Shanghai Jiao Tong University, Shanghai, China

## Abstract

MicroRNAs (miRNAs) are vital in the regulation of tumor progression and invasion. Dysregulation of miRNAs has been linked to the development of various types of human cancers, including non-small-cell lung cancer (NSCLC). However, the effect of miRNA-34a (miR-34a), a key regulator of tumor suppression, on the tumorigenesis of NSCLC has not been fully elaborated. Herein, we reveal that miR-34a is significantly downregulated in NSCLC tissues and cell lines, suggesting that miR-34a might function as a tumor suppressor in lung cancer. We also confirmed that epidermal growth factor receptor (EGFR) is a direct target of miR-34a, and our data reveal that siRNA knockdown of EGFR can inhibit cell proliferation, promote apoptosis and arrest cell-cycle progression. In addition, EGFR can reverse the suppressive function of miR-34a overexpression on proliferation and cell apoptosis. Furthermore, *in vivo* experiments demonstrated that miR-34a suppress tumor growth, both in the A549 xenograft model, as well as in the metastatic tumors in nude mice. Taken together, our findings suggest that miR-34a inhibits NSCLC tumor growth and metastasis through targeting EGFR.

## Introduction

Lung cancer is the leading cause of cancer-related deaths worldwide, and nearly 80% of lung cancer cases are currently classified as non-small-cell lung cancer (NSCLC).^1, 2^ Despite advances made in the treatment of NSCLC, the 5-year survival rate still remains very low (below 15%) due to disease recurrence or metastasis.^3^ The prevalence and lethality of this disease highlight the importance of investigating the mechanisms involved in the tumorigenesis of NSCLC, as well as prognosticating potential therapeutic targets for its treatment. Regarded as a cancer driver gene, epidermal growth factor receptor (EGFR) has an important role in the progression of NSCLC.^4, 5, 6^ EGFR affects numerous systems involved in oncogenesis, including DNA synthesis, cell cycle, cell proliferation, cell invasion and metastasis.^7, 8^ It has been proposed as an attractive and promising target for anticancer treatment.^9^ Somatic, activating mutations in EGFR have been identified in a significant minority of patients with NSCLC,^10^ and these mutations are associated with an ~70% response rate to some EGFR tyrosine kinase inhibitors (gefitinib, erlotinib and afatinib), with patients typically developing progression of disease after 9–12 months.

Some studies have demonstrated that in cancer therapies EGFR mutations can be regulated by microRNAs (miRNAs).^11^ MiRNAs are small non-coding RNAs consisting of 20–23 nucleotides that regulate gene expression by binding to the 3′-untranslated region (3′-UTR) of their target mRNAs.^12^ MiRNAs have been found to serve as tumor suppressors or oncogenes in various cancer types and clinical prognoses.^13, 14, 15^ In NSCLC, several deregulated miRNAs, such as miR-34a, let-7, miR-124 and miR-154, have been shown to regulate cell proliferation, apoptosis, migration and invasion.^16, 17, 18, 19^ They maybe suppress tumor or promote tumor growth.

MiR-34a, a member of miR-34 family, is located in the region of chromosome lp36.23.^20^ It is a tumor suppressor with lost or reduced expression levels.^21^ It is well known that miR-34a can significantly suppress tumor progression, such as in NSCLC,^18^ breast cancer,^22^ glioblastoma multiforme,^23^ head and neck squamous cell carcinoma^24^ and hepatocellular carcinoma.^25^ Therefore, exploring the function of miR-34a and the role of its possible target genes in NSCLC is essential to understanding the molecular mechanism of this miRNA in tumorigenesis. We make a hypothesis that miR-34a should regulate EGFR directly or EGFR signaling.

In this study we detected that miR-34a was downregulated in NSCLC patient samples and NSCLC cell lines. Furthermore, we demonstrated that EGFR was a direct target of miR-34a. We have identified that miR-34a acted as an important tumor suppressor in NSCLC with EGFR as a novel target, both *in vitro* and *in vivo*. Our results may provide a potential molecular therapeutic target for human NSCLC.

## Results

### MiR-34a is downregulated in NSCLC

**Figure 1:**
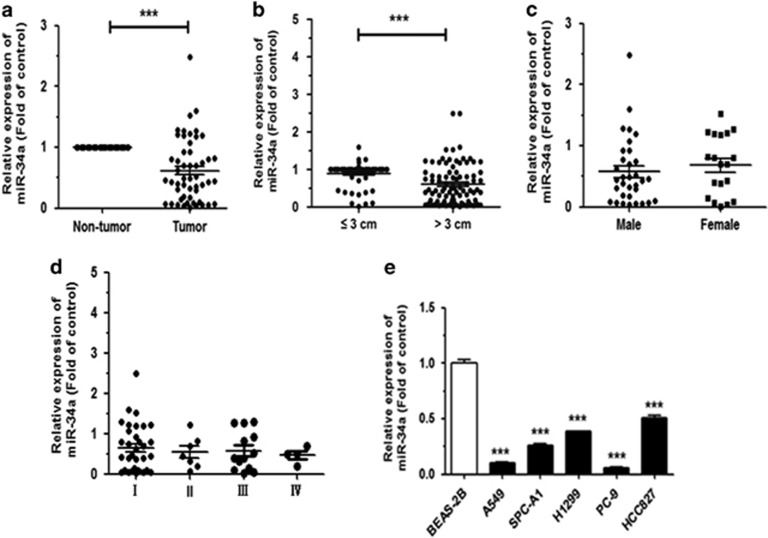
MiR-34a is downregulated in NSCLC tissues and human cell lines. (**a**) MiR-34a is downregulated in NSCLC tissues. (**b**–**d**) Expression of miR-34a as related to tumor size, sex and pathological stage. (**e**) The expression of miR-34a in the seven human NSCLC cell lines. BEAS-2B cells were used for the normal control comparison. ***P<0.001.

To evaluate the expression of miR-34a in NSCLC tissues, we performed quantitative real-time PCR (qRT–PCR) on 60 NSCLC patient samples and corresponding para-carcinoma tissues. This analysis revealed that the miR-34a expression level in NSCLC tissue samples was significantly lower than that in corresponding non-tumor tissues samples (Figure 1a). In these samples, the downregulation of miR-34a was closely related to tumor size (Figure 1b), but it was not associated with gender (Figure 1c) and pathological stage (Figure 1d). The miR-34a expression level in tissue samples from tumors >3 cm in size was significantly lower than that in tissue samples ⩽3 cm in size. We also analyzed the expression level of miR-34a in NSCLC cell lines. Results showed that compared with the normal bronchial epithelium cell, BEAS-2B, miR-34a was significantly downregulated in all five NSCLC cell lines (Figure 1e). These results suggested that downregulation of miR-34a may act as tumor suppressor gene in NSCLC.

### MiR-34a suppresses the proliferation and migration of NSCLC

**Figure 2:**
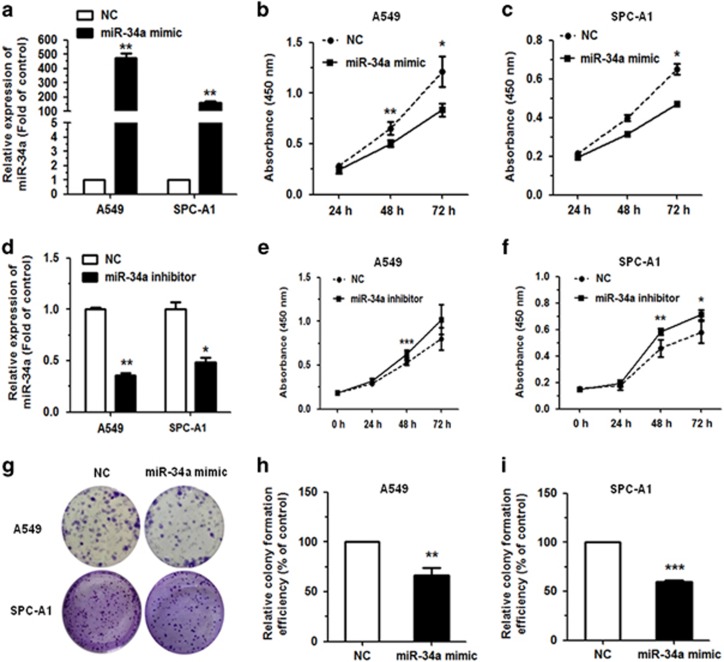
MiR-34a could inhibit cell proliferation in NSCLC cells. (**a**) Upregulation of miR-34a following transfection with 80 nmol miR-34a mimic in A549 and SPC-A1 cells. (**b**, **c**) The proliferation of A549 and SPC-A1 cell line, as measured by Cell Counting Kit-8 (CCK-8) assay, following transfection with miR-34a mimic. (**d**) Downregulation of miR-34a following transfection with 120 nmol miR-34a inhibitor in A549 and SPC-A1 cells. (**e**, **f**) The proliferation of A549 and SPC-A1 cell line, as measured by CCK-8 assay, following transfection with miR-34a mimic. (**g**–**i**) Colony formation assay in A549 and SPC-A1 cells transfected with miR-34a mimic. Each assay was performed in triplicate. *P<0.05, **P<0.01 and ***P<0.001.

The level of miR-34a expression was significantly upregulated in A549 and SPC-A1 cells transfected with miR-34a mimic, as compared with normal control (NC) mimic group. The cells were collected 48 h after transfection and miR-34a levels were detected by qRT–PCR analysis, resulting in ~400- and 100-fold increases for A549 and SPC-A1 cells, respectively (Figure 2a). Meanwhile, we transfected with miR-34a inhibitor, miR-34a level was downregulated when compared with NC mimic group (Figure 2d).

We examined the effect of miR-34a on the proliferation of NSCLC cells *in vitro* with Cell Counting Kit-8 (Dojindo, Tokyo, Japan) assay. Results showed that transfection of miR-34a mimic significantly inhibited the proliferation of the A549 (EGFR-wild type), SPC-A1 and HCC827 (EGFR-mutated) cell lines (Figures 2b and c; Supplementary Figure S1). While transfection with miR-34a inhibitor promoted the proliferation of the A549 and SPC-A1 cell lines (Figures 2e and f). This result indicated that miR-34a could inhibit the proliferation of NSCLC cell lines.

To further elucidate the function of miR-34a, we performed colony formation assay. Results showed that miR-34a could significantly inhibit colony formation in A549 or SPC-A1 cells with miR-34a mimic, when compared with the NC group (Figures 2g–i).

**Figure 3:**
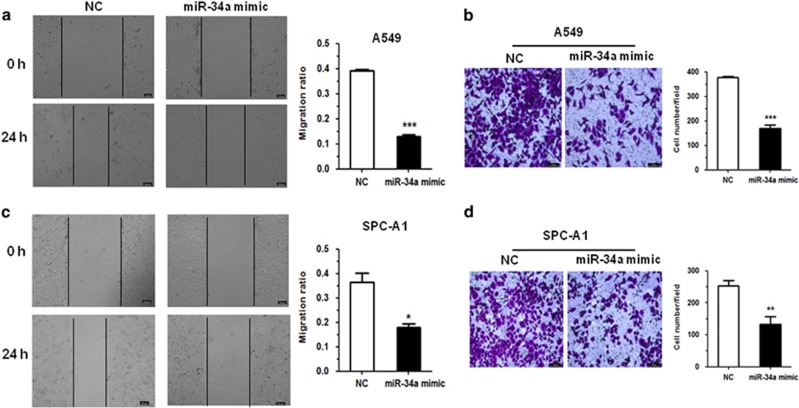
MiR-34a could inhibit migration in NSCLC cells. (**a**, **c**) A549 and SPC-A1 cells transfected with miR-34a mimic were subjected to wound healing assay and images were taken at 0 and 24 h. (**b**, **d**) Transwell migration assay performed after transfection of A549 and SPC-A1 cells with miR-34a mimic. The migrated cells were stained with crystal violet and photographed. Migrated cells were counted and analyzed. *P<0.05, **P<0.01 and ***P<0.001.

Furthermore, in A549 and SPC-A1 cells, miR-34a affected migration ability, a significant aspect of cancer progression. We performed wound healing and transwell assays. In the wound healing assay, A549 and SPC-A1 cells transfected with miR-34a mimic migrated toward the wound at a much slower rate than the NC group cells (Figures 3a and c). In the transwell assay, cells that had migrated from the serum-free medium in the top chamber of a two-chamber transwell cell culture plate to the lower chamber in 24 h were photographed and analyzed. Results showed that miR-34a could reduce the migration of A549 and SPC-A1 cells (Figures 3b and d). Together, these results indicated that miR-34a could significantly inhibit cell migration in the A549 and SPC-A1 cell lines.

### MiR-34a promotes cell apoptosis and arrests cell cycle

**Figure 4:**
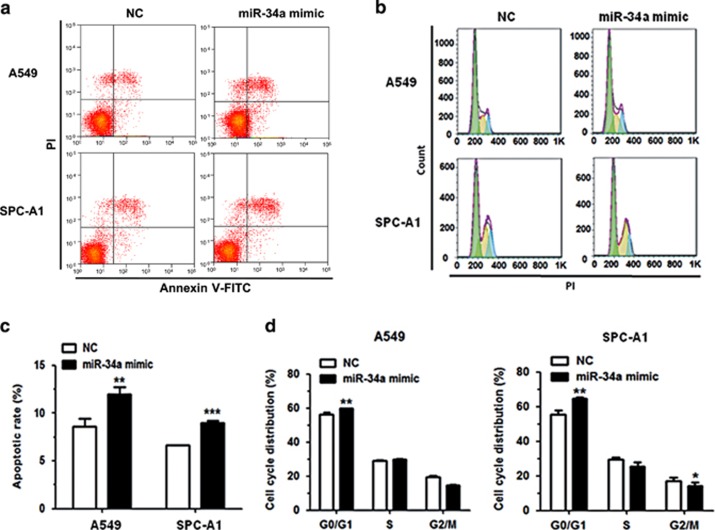
MiR-34a can promote cell apoptosis and impede cell-cycle progression in NSCLC cell lines. (**a**, **c**) The rate of apoptosis was analyzed by flow cytometry following transfection with miR-34a mimic in A549 and SPC-A1 cells. (**b**, **d**) The cell-cycle distributions of A549 and SPC-A1 cells transfected with miR-34a mimic were detected by flow cytometry. *P<0.05, **P<0.01 and ***P<0.001.

To investigate the underlying mechanism of action that miR-34a exerts on the inhibition of cancer cell proliferation, we performed flow cytometry after transfecting A549, SPC-A1 and HCC827 cells with miR-34a mimic and NC mimic. We analyzed the effect of miR-34a on cell apoptosis and cell-cycle progression. Results revealed that miR-34a overexpression significantly promoted apoptosis in A549, SPC-A1 and HCC827 cells, compared with the NC group (Figures 4a and c; Supplementary Figures S2a and b). With regard to cell cycle, transfection of miR-34a mimic in A549, SPC-A1 and HCC827 cells could partly inhibit cell-cycle progression (Figures 4b and d; Supplementary Figures S2c and d).

### EGFR is a direct target of miR-34a

**Figure 5:**
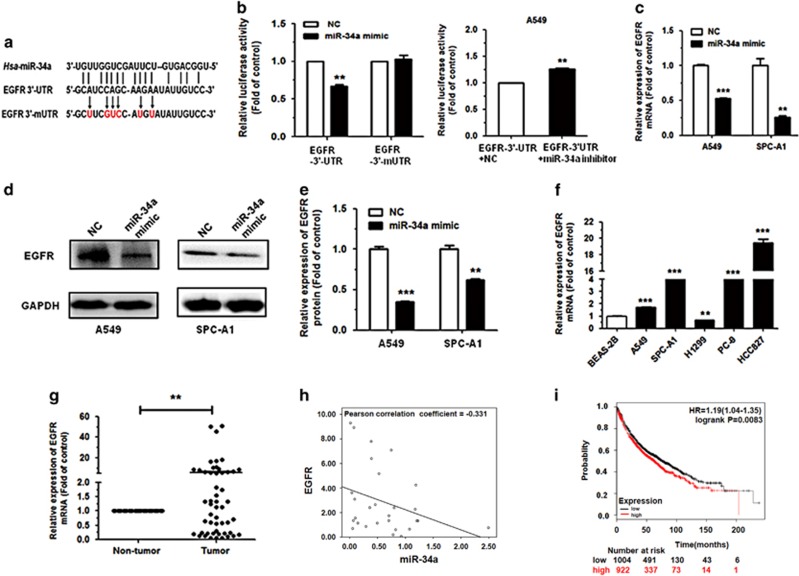
EGFR is a direct target of miR-34a. (**a**) EGFR WT 3′-UTR contains predicted miR-34a-binding site. The data show alignment of miR-34a with EGFR WT 3′-UTR and arrows indicate mutagenesis nucleotides. (**b**) Dual luciferase reporter assay. pGL3-EGFR WT 3′-UTR (EGFR-3′-UTR) and pGL3-EGFR mut 3′-UTR (EGFR-3′-mUTR) were co-transfected with miR-34a mimic in HEK293T cells (left). EGFR-3′-UTR were co-transfected with miR-34a inhibitor in A549 cells (right). Data displayed are relative firefly luciferase expression, normalized to Renilla luciferase expression. (**c**, **d**) The mRNA levels of EGFR were detected by qRT–PCR and protein levels were detected by western blot in A549 and SPC-A1 cells transfected with miR-34a mimic. (**e**) Densitometry was used to quantify the relative expression of EGFR proteins in A549 and SPC-A1 cells transfected with miR-34a mimic, after normalization to GAPDH. (**f**) The expression of EGFR in the five human NSCLC cell lines. BEAS-2B cells were used as the normal control for comparison. (**g**) The relative expression of EGFR mRNA from corresponding non-tumor tissues and tumor tissues as measured by qRT–PCR. 18S was used as an internal control. (**h**) There was a negative correlation between miR-34a and EGFR, according to the Pearson correlation coefficient. (**i**) The effect of EGFR expression levels on the overall survival of 1926 lung cancer patients was analyzed. Kaplan–Meier plots were generated using a Kaplan–Meier Plotter. *P<0.05, **P<0.01 and ***P<0.001.

To explore the molecular mechanism by which miR-34a contributes to the apoptosis and cell-cycle progression of NSCLC cells, we used miRWalk 2.0 (http://zmf.umm.uni-heidelberg.de/apps/zmf/mirwalk2/) and miRMap (http://mirmap.ezlab.org/) to predict potential targets, and identified EGFR as a potential target for miR-34a. To determine whether or not EGFR is a direct target of miR-34a, EGFR-wild type 3′-UTR (EGFR WT 3′-UTR) was cloned into the pGL3 vector (pGL3-EGFR WT 3′-UTR), downstream of the luciferase open reading frame. In addition, to validate target specificity, recombinant pGL3 vector with the mutation of EGFR-3′-UTR (pGL3-EGFR mut 3′-UTR) using a QuikChange Mutagenesis kit (Clontech, Shanghai, China) to construct (Figure 5a). There was a significant decrease of 50% in the relative luciferase activity of the report gene in human embryonic kidney 293T (HEK293T) cells co-transfected with EGFR-3′-UTR (pGL3-EGFR WT 3′-UTR), pRL vector and miR-34a mimic compared to the control (co-transfected with EGFR-3′-UTR, pRL vector and NC mimic). Inversely, co-transfection of miR-34a with EGFR-3′-mUTR (pGL3-EGFR mut 3′-UTR) resulted in no significant change in luciferase activity, supporting miRNA/target 3′-UTR specificity (Figure 5b). We also co-transfected EGFR-3′-UTR, pRL vector and miR-34a inhibitor, as well as the control (co-transfected with EGFR-3′-UTR, pRL vector and NC inhibitor) into A549 cell line. Results showed that the relative luciferase activity of the report gene in A549 cell was increased compared to the control (Figure 5b).

To further investigate whether miR-34a reduced EGFR expression at both the transcriptional and translational levels in NSCLC cells, we performed qRT–PCR and western blotting to determine the mRNA and protein levels of EGFR. Results showed that the levels of EGFR mRNA and protein expression were significantly decreased following transfection with miR-34a mimic in both A549 and SPC-A1 cells, compared with the NC mimic group (Figures 5c–e). The EGFR expression in A549, SPC-A1, PC-9 and HCC827 was much higher than that of the control (BEAS-2B; Figure 5f).

To investigate whether or not EGFR expression was inversely correlated with miR-34a in NSCLC tissues, we evaluated the mRNA expression of EGFR in the 60 primary NSCLC tumors and non-tumor tissues using qRT–PCR. Among the pairs of tissues, we found that the level of EGFR expression was upregulated in tumor tissues, when compared with their non-tumor tissues (Figure 5g). In addition, there was a negative correlation between the relative EGFR expression and miR-34a in the NSCLC tissues samples, according to statistical analysis using the Pearson correlation coefficient (Figure 5h).

To analyze the effect of EGFR expression in lung cancer patients, we generated a Kaplan–Meier survival curve of NSCLC patients with low or high EGFR expression using the Kaplan–Meier Plotter online database (www.kmplot.com/analysis; Figure 5i). Among the 1926 cases, we found that NSCLC patients with high EGFR expression had lower survival rates.

**Figure 6:**
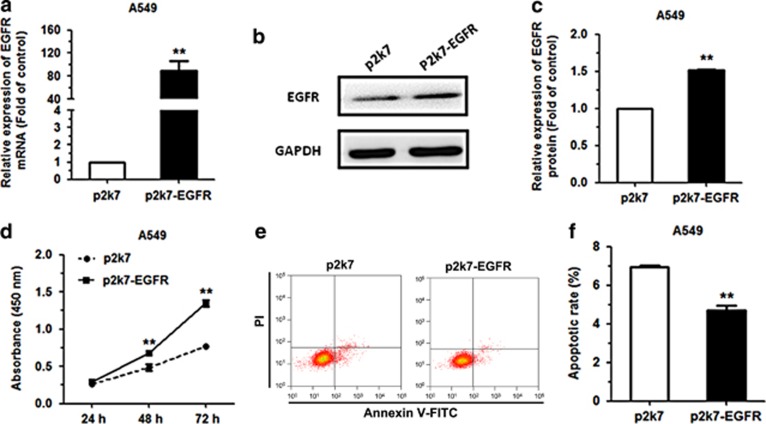
EGFR can rescue the effects of miR-34a on NSCLC cell lines. (**a**–**c**) The levels of EGFR mRNA and protein in miR-34a-stably overexpressing (pLenti-miR-34a) and negative control (pLenti) A549 cells following transfection with EGFR expression vector (p2k7-EGFR) and negative control (p2k7). (**d**) The proliferation of A549 (pLenti-miR-34a/pLenti) cells following transfection with p2k7-EGFR and p2k7, as measured by Cell Counting Kit-8 assay. (**e**, **f**) The apoptosis rates of A549 (pLenti-miR-34a/pLenti) cells following transfection with p2k7-EGFR and p2k7 were analyzed by flow cytometry. *P<0.001.

To investigate whether the effects of miR-34a was mediated through EGFR, we transfected EGFR expression vector (p2k7-EGFR) and negative control (p2k7) into A549 cell line, which stably overexpressing miR-34a (pLenti-miR-34a) and negative control (pLenti). To investigate whether the effects of miR-34a was mediated through EGFR, The pLenti and the pLenti-miR-34a A549, positive cells for green fluorescence was almost 99% (Supplementary Figure S3a). The level of miR-34a expression was upregulated 26-fold in A549 pLenti-miR-34a, as compared with pLenti groups (Supplementary Figure S3b). The mRNA and protein expression of EGFR were also increased following transfection with pLenti-miR-34a in A549 cell, compared with the NC group (Figures 6a–c). We also analyzed the effect of miR-34a overexpression on NSCLC proliferation and cell apoptosis. Results revealed that EGFR reverse the suppressive function of miR-34a overexpression on proliferation and cell apoptosis in A549 cell, compared with the NC group (Figures 6d and f). In conclusion, these data suggested that the effects of EGFR promote NSCLC by increasing cell proliferation and inhibiting apoptosis.

### Downregulation of EGFR inhibits cell proliferation, promotes cell apoptosis and induces cell-cycle progression in NSCLC cell lines

**Figure 7:**
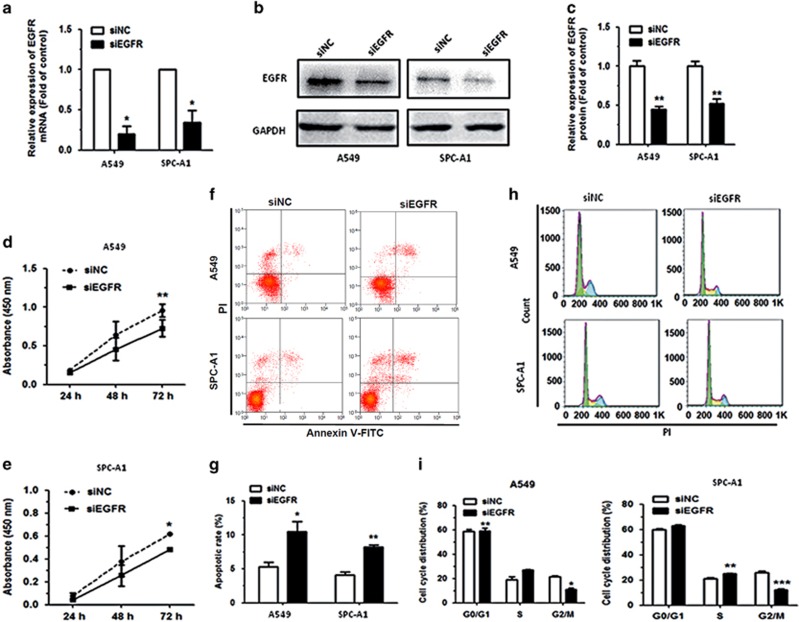
Downregulation of EGFR can inhibit cell proliferation, promote cell apoptosis and impede cell-cycle progression in NSCLC cell lines. (**a**, **b**) The levels of EGFR mRNA and protein in A549 and SPC-A1 cells transfected with siEGFR were measured by qRT–PCR and western blot, respectively. (**c**) Densitometry was used to quantify the relative expression of EGFR protein, after normalization to GAPDH, in A549 and SPC-A1 cells transfected with siEGFR. (**d**, **e**) Proliferation of A549 and SPC-A1 cells transfected with siEGFR was determined by Cell Counting Kit-8. (**f**, **g**) The rate of apoptosis of A549 and SPC-A1 cells transfected with siEGFR was analyzed by flow cytometry. (**h**, **i**) The cell-cycle distributions of A549 and SPC-A1 cells transfected with siEGFR were detected by flow cytometry. *P<0.05, **P<0.01 and ***P<0.001.

We knocked down EGFR using siRNA to downregulate EGFR expression and investigate the effects on cell proliferation, cell apoptosis and cell-cycle progression in NSCLC cell lines, A549 and SPC-A1. Our results revealed that the expression levels of EGFR mRNA and protein were significantly downregulated by more than 50% in NSCLC cells transfected with EGFR siRNA (siEGFR) compared with the NC siRNA (siNC)-transfected cells (Figures 7a–c). Results further revealed that the proliferation of cells transfected with siEGFR was significantly suppressed, compared with the siNC-transfected cells (Figures 7d and e). We next analyzed the effects of transfection with siEGFR on apoptosis and found it yielded a significant increase in the rate of apoptosis in A549 and SPC-A1 cells, compared with the siNC-transfected cells (Figures 7f and g). Furthermore, flow cytometric analysis of cell cycle showed that the proportion of cells at G0/G1 phase was increased in cells transfected with siEGFR when compared with the siNC-transfected cells (Figures 7h and i).

Collectively, these results demonstrated that EGFR knockdown exhibited a similar phenotype as miR-34a upregulation in NSCLC cells, and that the tumor suppressive function of miR-34a is mediated partly by the downregulation of EGFR.

### MiR-34a suppresses tumor growth in A549 xenograft and metastatic tumors

**Figure 8:**
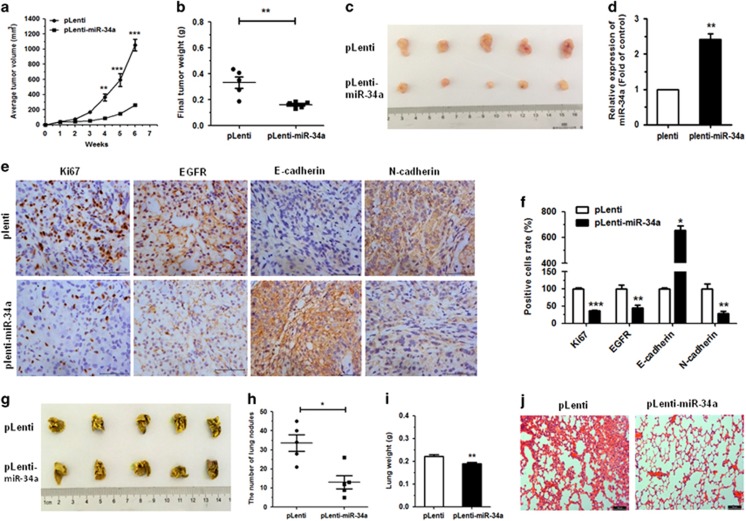
Upregulation of miR-34a-inhibited tumor growth and metastases *in vivo*. (**a**) pLenti-miR-34a A549 cells and control cells (pLenti) were injected into nude mice by subcutaneous or tail vein injection. Tumor volumes were measured weekly and growth curves were generated. (**b**) Tumor weights of mice 6 weeks after subcutaneous injection. (**c**) Tumor images are displayed. (**d**) The expression of miR-34a was detected by qRT–PCR in the mouse tumor tissues induced by pLenti-miR-34a A549 cells and pLenti cells. (**e**, **f**) The expression of Ki67, EGFR, E-cadherin and N-cadherin in tumor tissues was measured by immunohistochemistry. (**g**) The lungs of mice with metastasis nodes are displayed. (**h**, **i**) The numbers of lung nodes and weight of lungs of mice induced by pLenti-miR-34a A549 cells and pLenti cells are displayed. (**j**) Histopathology of metastases with hematoxylin and eosin staining (original magnification × 100). *P<0.05, **P<0.01 and ***P<0.001.

On the basis of the tumor suppressive role of miR-34a *in vitro*, we further examined the role of miR-34a *in vivo*, to assess its therapeutic potential. Nude mice were injected subcutaneously with miR-34a-stably overexpressing (pLenti-miR-34a) A549 cells (5 × 10^6^) or control (pLenti), and the subsequent tumors were assessed after 6 weeks. The A549 xenografts displayed significant inhibition of tumor growth in pLenti-miR-34a group, compared to the control (Figure 8a). The pLenti-miR-34a cells also exhibited obvious reduction in both tumor size and weight at 6 weeks post implantation (Figures 8b and c). Furthermore, a significant upregulation in the expression of miR-34a was observed in tumor tissues from the pLenti-miR-34a group, when compared to the controls (Figure 8d). We also measured the expression of Ki67, EGFR, E-cadherin and N-cadherin in the xenograft tumor tissues using immunohistochemistry. Results showed a significant downregulation of Ki67 and EGFR in the tumor tissues stably pLenti-miR-34a, accompanied by an induction of E-cadherin expression, but a decrease in the expression of N-cadherin (Figures 8e and f). These data indicated that miR-34a could inhibit tumor growth *in vivo*, complementing the results of our functional *in vitro* studies.

To ascertain whether or not miR-34a effected tumor metastasis *in vivo*, A549 cells (5 × 10^6^) pLenti-miR-34a or pLenti were injected into the tail vein of nude mice. Six weeks after tail vein injection, we observed a marked effect on both the number of the nodes eventually formed, as well as the weight of the lungs with metastases. The pLenti cells generated fine and scattered metastatic nodes, while the pLenti-miR-34a cells resulted in massive and confluent metastatic nodes (Figures 8g and h). Statistical analysis showed that the weight of the lungs from mice with metastatic nodes in the pLenti-miR-34a cell group had shrunk about 0.85-fold compared with those in the control cell group (Figure 8i). Simultaneously, hematoxylin and eosin staining under light microscopy was used to observe pathological changes in the bilateral lungs. The tumor nests derived from control cells exhibited a large area of lung tissue destruction and/or necrosis, whereas pLenti-miR-34a cells formed fewer and smaller tumor nests (Figure 8j).

## Discussion

Our previous studies have confirmed that the miRNAs, miR-181a-5p, miR-146a-5p, miR-32, miR-34a and miR-486-5p, have important roles in the progression of NSCLC.^18, 26, 27, 28^ These findings demonstrated that altered miRNA expression might be related to the tumorigenesis of lung cancer.

It has been suggested that specific miRNA could act as a molecular diagnostic tool for lung cancer,^29, 30^ and miRNA-based therapy could potentially be a rational approach for the therapeutic targeting of EGFR.^31^ Recently, a number of miRNAs such as miR-200a,^32^ miR-27a/27b,^33, 34^ miR-133a,^35^ miR-134,^36^ miR-143,^37^ miR-145^38^ and miR-146a^39^ have been demonstrated to target EGFR directly. These studies on the regulation network of EGFR-miRNAs highlight the possibility that in addition to tyrosine kinase inhibitors and classical monoclonal antibodies for EGFR-targeted therapies, miRNA-based therapy could be utilized to target EGFR.^31^ Moreover, study has shown that the therapeutic potential of miR-34a delivery in combination with radiotherapy may represent a novel strategy for treating NSCLC.^30^ Meanwhile, there are many verified and predicted targets of miR-34a (Supplementary Table S4). Here we identified miR-34a as an EGFR-targeting miRNA.

To date, miR-34a is best understood as a more and more anticancer miRNA, acting primarily on cell-cycle arrest, differentiation, senescence and apoptosis,^20, 40^ and it is significant to note that a liposomal formulation of miR-34a (MRX34) has become the first miRNA to reach phase I clinical trials.^20, 41, 42^ Recently, studies have further discovered that ectopic overexpression of miR-34a results in the inhibition of some solid tumors, including prostate cancer,^43^ colon cancer,^44, 45^ NSCLC,^46^ endometrial carcinoma,^47^ hepatocellular carcinoma^48^ and osteocarcinoma.^49^ MiR-34a has the ability to control the expression of a gene family through combining bioinformatic approaches with experimental validation.^50^ Numerous human disease pathogenesis have related with the change expression of miRNA.^51^

Our previous study verified that in NSCLC, overexpression of miR-34a inhibits proliferation and promotes apoptosis *in vitro*.^18^ This result may suggest the potential for miR-34a as a tumor suppressor in NSCLC. Besides, the mechanism of miR-34a in blocking NSCLC tumor growth deserves more exploration. Our present study demonstrated that miR-34a expression was reduced in NSCLC, when compared with the non-tumor tissues. Moreover, we verified that miR-34a inhibits proliferation of lung cancer cells by inducing cell apoptosis and cell-cycle arrest. Furthermore, since miR-34a inhibits *in vitro* proliferation of NSCLC cells, we explored miR-34a’s potential for *in vivo* tumor suppression. We have demonstrated that in the NSCLC cell lines, A549, SPC-A1 and HCC827 (EGFR-mutated), miR-34a acted as a tumor suppressor through the direct targeting of EGFR.

**Figure 9:**
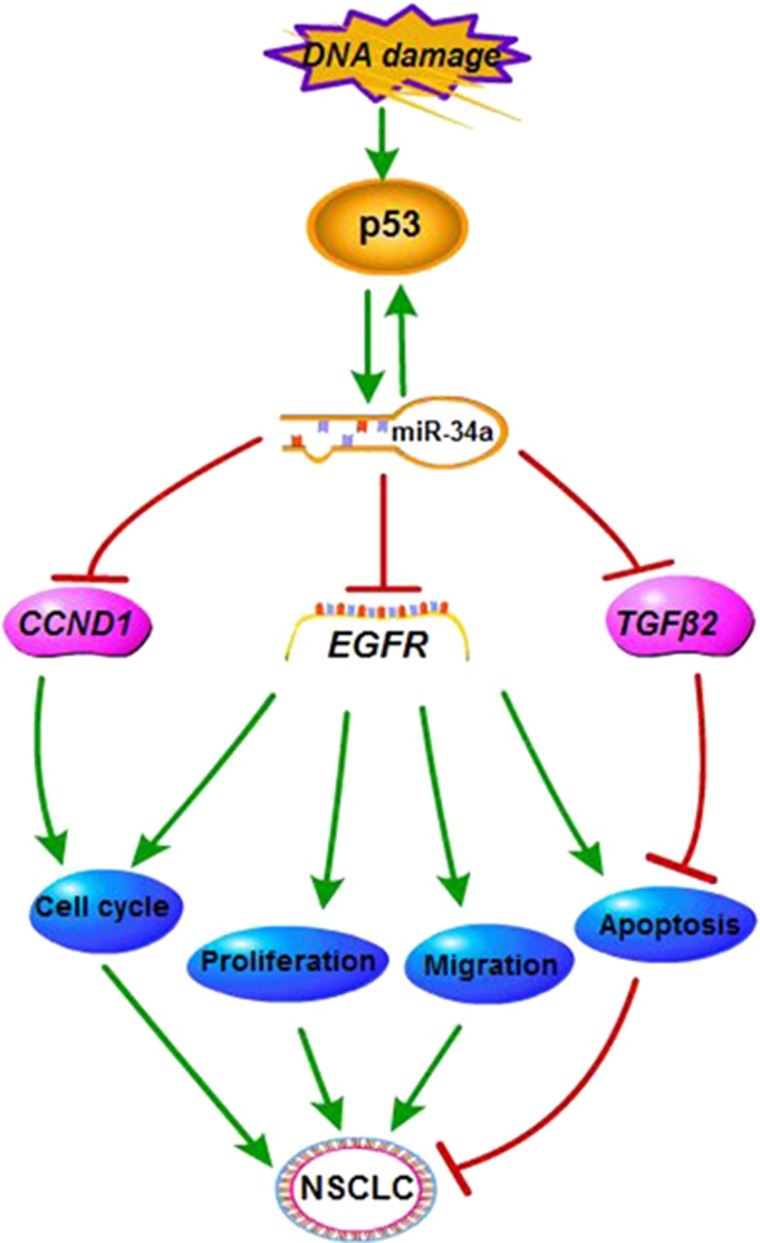
The regulatory network of miR-34a in NSCLC. We present that miR-34a has the role of tumor suppressor in NSCLC through the downregulation of EGFR.

Tumor progression and development is precisely regulated by several subsets of genes that act by either activating oncogenes or silencing tumor suppressor genes.^52^ Tumor suppressor genes can negatively regulate cell proliferation by inhibiting growth and inducing cell apoptosis. Our xenograft data suggested that suppression of tumor growth and metastasis occurs through the inhibition of the proliferation and the induction of apoptosis of the tumor cells, indicating the potential for miR-34a as a strategy for EGFR-targeted therapy. Moreover, based on prior studies in our lab,^18, 26, 27, 28^ we concluded that miR-34a could be involved in the regulation of several target mRNAs, and also be closely related to the signaling pathways for Kras and nuclear factor-κB. As a tumor suppressor within this regulation network, miR-34a can affect the proliferation, migration, apoptosis and cell cycle of NSCLC cells (Figure 9). Our findings have provided the opportunity to understand the mechanism of oncogenesis miRNAs have on the regulation of lung cancer.

In conclusion, our study shows that miR-34a inhibits NSCLC growth by targeting EGFR. Therefore, miR-34a and EGFR might be promising molecular targets not only for the treatment of NSCLC but also as a useful and novel prognostic or progression marker for NSCLC.

## Materials and methods

### Clinical tissue samples

Tissue samples were obtained from, and with approval from the ethics committee of, the Shanghai Chest Hospital, affiliated with Shanghai Jiao Tong University. Details of all samples used in this paper are listed in Supplementary Table S1.

### Cell culture

SPC-A1, HCC827, BEAS-2B and HEK293T cells were obtained from the Cell Bank, China Academy of Sciences (Shanghai, China). A549, H1299 and PC-9 cells were purchased from the American Type Culture Collection (Manassas, VA, USA). The BEAS-2B cell line was isolated from normal human bronchial epithelium.

The NSCLC cell lines, H1299 and HCC827 were cultured in either RPMI-1640 medium or Dulbecco’s modified Eagle’s medium (DMEM, Gibco, Gaithersburg, MD, USA). BEAS-2B cells were cultured in LHC-9 medium, and A549, SPC-A1, PC-9 and HEK293T cells were cultured in DMEM. All media were supplemented with 10% fetal bovine serum (HyClone Laboratories, Logan, UT, USA), and an antibiotic cocktail of 100 U/ml penicillin and 100 μg/ml streptomycin (Gibco). Cell culture was carried out at 37 °C in a 5% CO_2_ humidified environment.

### RNA extraction and qRT–PCR

Total RNA was extracted with TRIzol reagent (Bio Basic Inc., Toronto, ON, Canada), Reverse transcription was performed with the QuantiMir cDNA Kit (Takara, Dalian, China) and PrimeScriptTM RT reagent Kit (Takara); quantitative PCR was carried out with SYBR Premix Ex Taq (Takara) according to the manufacturer’s protocols. U6 snRNA and 18S rRNA were used as the endogenous controls for miRNA and mRNA. Results were expressed using the relative quantification (2^-ΔΔCt^) method. Primer sequences are shown in Supplementary Table S3.

### Cell transfection

MiR-34a mimic, NC mimic, miR-34a inhibitor, NC inhibitor and siRNA were purchased from Ruibo Company (Guangzhou, China).

Cells were transfected with 80 nm of chemically synthesized miR-34a mimic, 120 nm miR-34a inhibitor or 100 nm siEGFR-1 (Supplementary Table S2) using Lipofectamine 2000 (Thermo, Life Technologies, New York, NY, USA), according to the manufacturer’s instructions. After 24 h post transfection, cells were used for subsequent experiments including assays for cell proliferation, migration, apoptosis and cell-cycle analysis, qRT–PCR and western blotting.

### Cell proliferation assay

Cell proliferation was determined with Cell Counting Kit-8 assay. Briefly, cells were plated in a 96-well plate at a density of 2 × 10^3^ cells per well and incubated at 37 °C in a 5% CO_2_ humidified environment. Cell Counting Kit-8 was added and cells were returned to incubate for 1–4 h. Light absorbance at 450 nm was measured daily with a microplate reader. Experiments with triplicates were performed independently at least thrice.

### Colony formation assay

Cells were plated at 300 cells per 35 mm tissue culture dish and incubated for 2 weeks at 37 °C in a 5% CO_2_ humidified environment. Colonies were then fixed with methanol, stained with crystal violet (0.5% w/v) and counted. Experiments with duplicates were performed independently at least thrice.

### Cell migration assay

A549 and SPC-A1 were seeded at 2 × 10^5^ cells per well in 24-well plate and allowed to reach confluence. A single-scratch wound was introduced through the middle of each well with a sterile pipette tip. Cell migration across the margins was assessed and photographed after 24 h.

Cell transwell assay was performed using 24-well transwells (6.5 mm pore size, Corning Life Sciences, Manassas, VA, USA). A549 and SPC-A1 cells were transfected with 80 nm miRNA mimic. After 24 h, were added to the upper chamber of each migration well in 100 μl fetal bovine serum-free medium, and 500 μl DMEM with 10% fetal bovine serum was added to the lower part of the chamber and migration was allowed to occur for 24 h. The cells on the filter surface were fixed with methanol, stained with crystal violet and photographed with a phase-contrast inverted microscope. Experiments with triplicates were performed independently at least thrice.

### Cell apoptosis analysis

Cell apoptosis analysis was performed as previously described.^18^ An Annexin V-fluorescein isothiocyanate apoptosis detection kit (BD Pharmingen, San Diego, CA, USA) was used according to the manufacturer’s instruction to determine the level of cell apoptosis. A549 and SPC-A1 cells were resuspended in 1 × binding buffer solution with Annexin V-fluorescein isothiocyanate and propidium iodide, and then incubated at room temperature for 15 min in the dark. Apoptotic cells were analyzed using a MoFlo XDP flow cytometer (Beckman Coulter, Inc., Brea, CA, USA). Experiments with triplicates were performed independently at least thrice.

### Cell-cycle analysis

Cell-cycle analysis was performed as previously described.^27^ In brief, 1 × 10^5^ cells were collected and fixed in 75% ethanol overnight at −20 °C. The cells were then collected, treated with RNase A (100 ng/ml) for 30 min and stained with propidium iodide (50 ng/ml) for 15 min. After staining, samples were analyzed for cell-cycle distribution with a MoFlo XDP flow cytometer (Beckman Coulter). Data were analyzed using Flow Jo software (Treestar Inc., Brea, CA, USA). Experiments with duplicates were performed independently at least thrice.

### Luciferase reporter assay

3′-UTR luciferase plasmids were constructed as follows: the 3′-UTR of EGFR containing the predicted binding site of miR-34a was cloned into the pGL3 vector (Promega, Madison, WI, USA) and designated as EGFR-3′-UTR. EGFR-3′-mUTR plasmid containing the mutated binding site was also constructed. Site-directed mutagenesis was performed by quick-change PCR with mutated primer pairs and Pfu polymerase (Takara). Recombinant expression vectors were confirmed by sequencing (Sangon Biotech, Shanghai, China). Primer sequences are listed in Supplementary Table S3.

miRWalk 2.0 (http://zmf.umm.uni-heidelberg.de/apps/zmf/mirwalk2/) and miRMap (http://mirmap.ezlab.org/) were used to predict the target genes of miR-34a. According to miRWalk 2.0, highly conserved predictions were included as the potential targets.

Luciferase reporter assay was performed as previously described.^18, 27^ In brief, HEK293T cells were cultured in 24-well plate and transiently co-transfected with 200 ng of luciferase vector EGFR-3′-UTR or EGFR-3′-mUTR, and a final concentration of 100 nm of miR-34a mimic or NC mimic, with 20 ng of plasmid expressing the renilla luciferase gene (pRL, Promega) as a control for transfection efficiency. After 48 h post transfection, cells were lysed and luciferase activity was assayed with an Orion II Microplate Illuminometer (Titertek-Berthold, South San Francisco, CA, USA). Relative activities were expressed as the fold-change in luciferase activity following normalization to renilla luciferase activity.

A549 cells were cultured in 24-well plate and transiently co-transfected with 200 ng of luciferase vector EGFR-3′-UTR and a final concentration of 100 nm of miR-34a inhibitor or NC inhibitor, with 20 ng of plasmid expressing the renilla luciferase gene (pRL, Promega) as a control for transfection efficiency. Relative activities were expressed as the fold-change in luciferase activity following normalization to renilla luciferase activity.

### Protein extraction and western blot analysis

Western blot analysis was performed as previously described.^18, 27^ In brief, total protein was extracted from the cells using RIPA lysis buffer (CWBIO, Beijing, China) and quantified with a Protein BCA Assay Kit (Bio-Rad, Hercules, CA, USA). The protein was then separated by SDS–polyacrylamide gel electrophoresis and transferred to a polyvinylidene difluoride membrane (Millipore Corporation, Billerica, MA, USA). The membrane was then blocked with 5% powdered milk at room temperature for 1 h, followed by incubation with rabbit anti-EGFR (4267 S) and anti-GAPDH (14C10) antibodies (1:1000, Cell Signaling Technology, Danvers, MA, USA) overnight at 4 °C. After washing and incubation with a goat-anti-rabbit secondary antibody conjugated to horseradish peroxidase (1:1000, Cell Signaling Technology), protein bands were detected with a chemiluminescent horseradish peroxidase substrate (Millipore) and imaged with an E-Gel Imager (Bio-Rad, Hercules, CA, USA).

### Lentivirus construction and infection

Lentivirus construction and infection performed as previously described.^53^ In brief, pri-miR-34a sequence was digested with *Bam*HI and *Xho*lI, then cloned into the pLenti vector (Invitrogen, Carlsbad, CA, USA), formed pLenti-miR-34a. pLenti-miR-34a or pLenti vector was co-transfected into HEK293T with psPAX2 and pMD2G. Viral particles were collected 48 and 72 h later, centrifuged them together at 4000 r.p.m. for 5 min at 4 °C, then filtered with 0.45 μm filter. A549 cells were then infected with the viral particles, transfected with pLenti-miR-34a or pLenti and were sorted for green fluorescence via flow cytometry. The cells were transfected with pLenti-miR-34a or pLenti and sorted for green fluorescence via flow cytometry.

### EGFR rescue assay

pLenti A549 and pLenti-miR-34a A549 were transfected with 1000 ng/ml EGFR expression vector p2k7 or p2k7-EGFR using Lipofectamine 2000. After 24 h post transfection, cells were used for qRT–PCR, western blotting, then for cell proliferation and apoptosis analysis. Experiments with triplicates were performed independently at least thrice.

### Tumor xenograft assay and metastatic assay

Female nude mice were purchased from the SLRC Laboratory Animal Center (Shanghai, China) and maintained under specific-pathogen-free conditions. To establish the subcutaneous tumor xenograft model, 6–8 weeks’ mice were randomly assigned into two groups (five mice per group), and each nude mice was injected subcutaneously in the right flank with 5 × 10^6^ A549 cells (resuspended in 100 μl DMEM medium) pLenti-miR-34a or pLenti. Tumor diameters were measured weekly with calipers, and tumor volumes were calculated using the following formula: volume=length × width^2^/2. Upon conclusion of the experiment, the mice were killed and primary tumors were excised and weighed. To investigate the metastatic ability, mice were injected with 5 × 10^6^ cells (resuspended in 200 μl DMEM medium) via the tail vein, and 6 weeks following the injection, the mice were killed and lung tissues were isolated. Evaluation of lung tissue weights was used to quantify metastasis. Six tumor tissues were subjected to serial sectioning and immunohistochemistry. Six lung tissues were subjected to serial sectioning and then hematoxylin and eosin staining. Pathological changes were observed under a light microscope. All experimental protocols were approved by the Institutional Animal Care and Use Committee of Shanghai University (Shanghai, China). All efforts were made to minimize suffering.

### Immunohistochemistry

Tumor growth was assessed by immunohistochemical staining of Ki67 to quantitate the growth index, N-cadherin and E-cadherin to quantitate the migration index. Target gene for miR-34a, *EGFR*, was also measured by immunohistochemistry. Tumor biopsies were fixed with formalin, embedded in paraffin and cut into sections of about 4 μm. Samples were then deparaffinized and dehydrated with xylene and graded alcohols, and subsequently rehydrated with demineralized water. Immunohistochemistry was performed using microwave pre-treatment of slides for antigen retrieval. Primary antibodies against Ki67, EGFR, N-cadherin and E-cadherin (1:500, Cell Signaling Technology) were applied, together with goat-anti-rabbit horseradish peroxidase-conjugated antibodies, and proteins were visualized *in situ* with a 3, 3′-diaminobenzidine reaction solution.

### Statistical analysis

Results are expressed as the group means±s.e.m. and analyzed using (GraphPad Prism 5 software, La Jolla, USA), using *t*-tests for two-group comparisons. Differences were considered statistically significant when *P*<0.05.

## PUBLISHER’S NOTE

Springer Nature remains neutral with regard to jurisdictional claims in published maps and institutional affiliations.

## Supplementary Material

Supplementary Figure S1

Supplementary Figure s2
