# Supplementary figures and images for: MicroRNA-34a/EGFR axis plays pivotal roles in lung tumorigenesis
Source: Oncogenesis. 2017 Aug 21;6(8):e372–. doi: 10.1038/oncsis.2017.50 (PMC5608916; doi:10.1038/oncsis.2017.50)

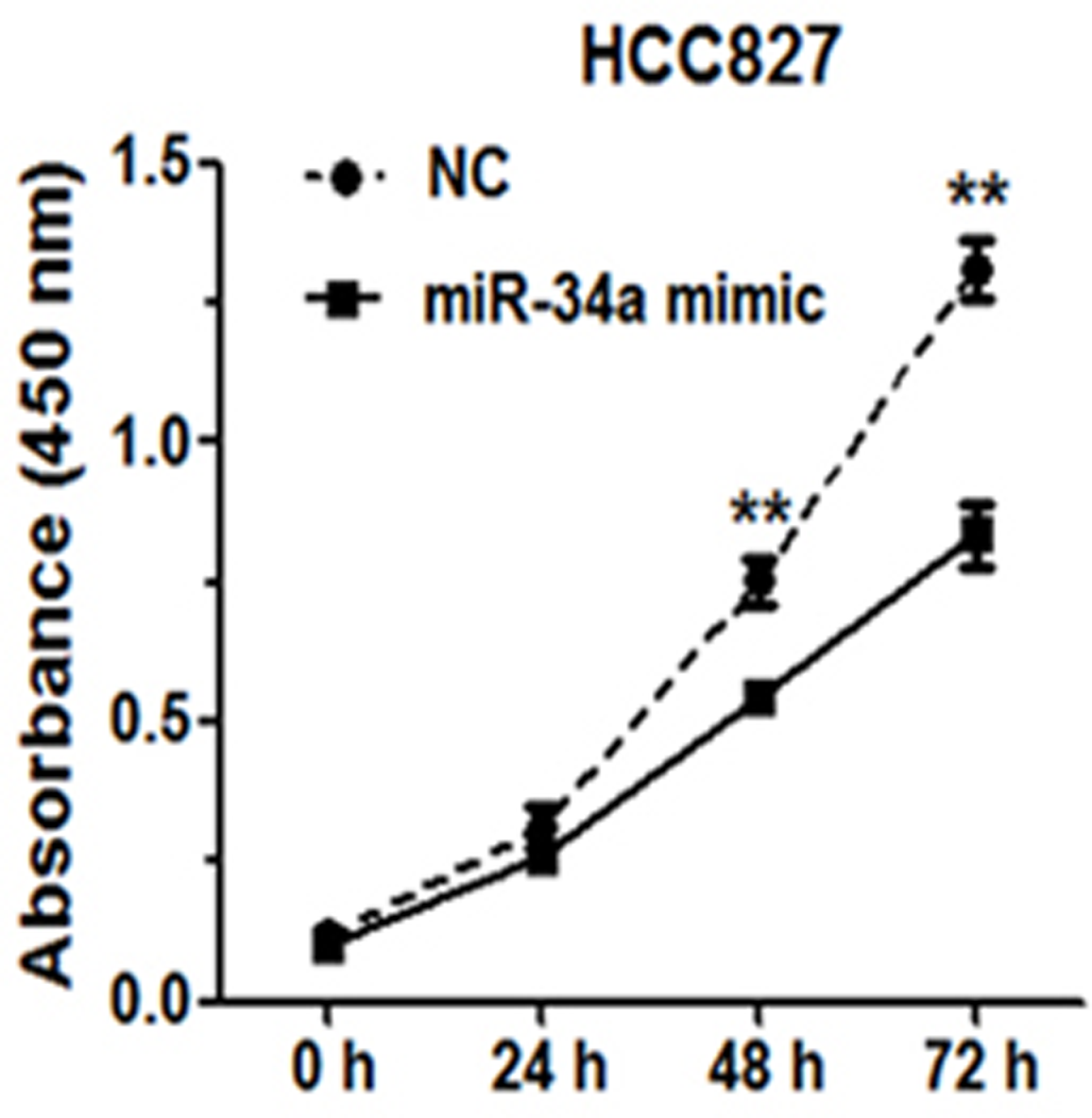

Supplement: Supplementary Figure 1 [file oncsis201750x1.tif]

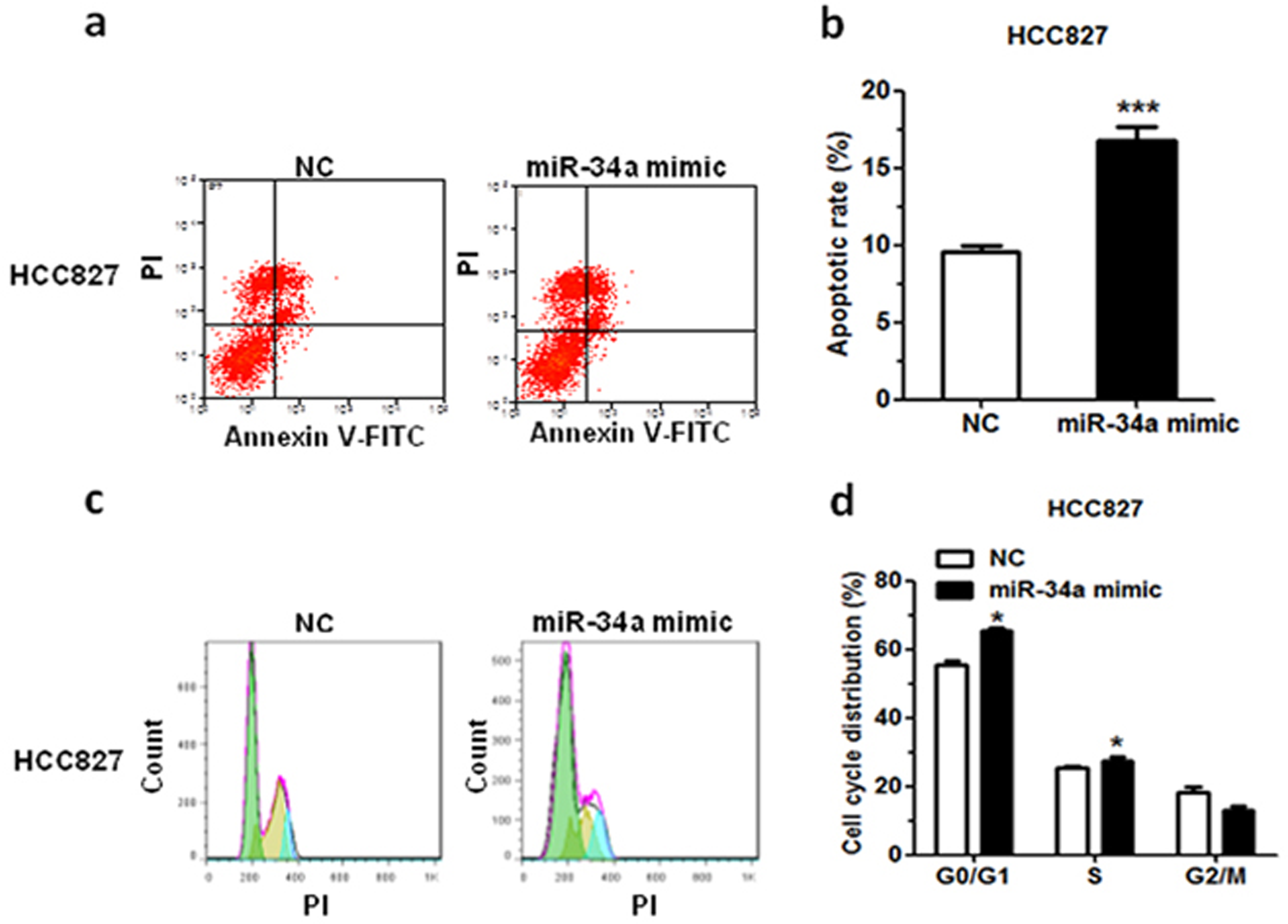

Supplement: Supplementary Figure 2 [file oncsis201750x2.tif]

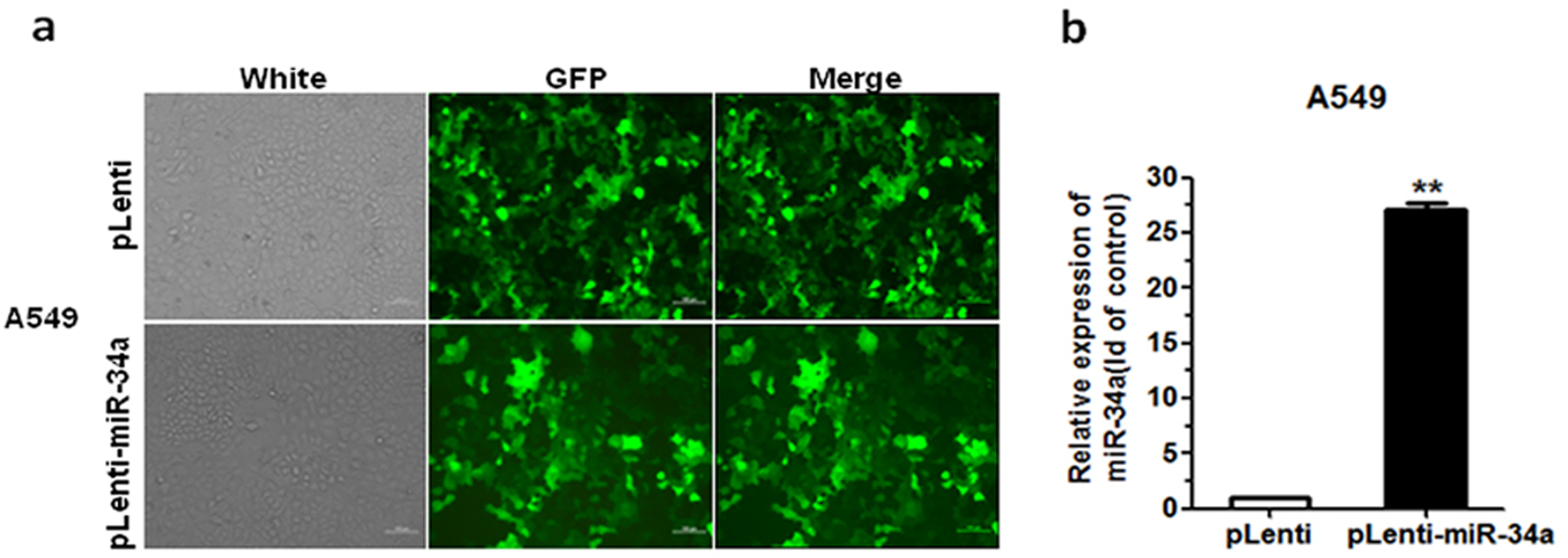

Supplement: Supplementary Figure 3 [file oncsis201750x3.tif]
