# Supplementary material for: MicroRNA-34a/EGFR axis plays pivotal roles in lung tumorigenesis
Source: Oncogenesis. 2017 Aug 21;6(8):e372–. doi: 10.1038/oncsis.2017.50 (PMC5608916; doi:10.1038/oncsis.2017.50)
Supplement: Supplementary Figure S1 [file oncsis201750x4.docx]

**Supplementary**

**Figure S1. MiR-34a could inhibit HCC827 proliferation.**

The proliferation of HCC827 cells as measured by CCK-8 assay, following transfection with miR-34a mimic. ***P*<0.01.
