# Supplementary material for: MicroRNA-34a/EGFR axis plays pivotal roles in lung tumorigenesis
Source: Oncogenesis. 2017 Aug 21;6(8):e372–. doi: 10.1038/oncsis.2017.50 (PMC5608916; doi:10.1038/oncsis.2017.50)
Supplement: Supplementary Figure s2 [file oncsis201750x5.docx]

**Supplementary**

**Figure S2. MiR-34a can promote HCC827 apoptosis and impede cell-cycle progression.**

**(a, b)** The rate of apoptosis was analyzed by flow cytometry following transfection with miR-34a mimic in HCC827 cells.

**(c, d)** The cell cycle distributions of HCC827 cells transfected with miR-34a mimic were detected by flow cytometry. **P*<0.05 and ****P*<0.001.
