# Supplementary material for: MicroRNA-34a/EGFR axis plays pivotal roles in lung tumorigenesis
Source: Oncogenesis. 2017 Aug 21;6(8):e372–. doi: 10.1038/oncsis.2017.50 (PMC5608916; doi:10.1038/oncsis.2017.50)
Supplement: Supplementary Figure 3 [file oncsis201750x6.docx]

**Supplementary**

**Figure S3. The expression level of miR-34a is significantly upregulated in pLenti-miR-34a A549 cells.**

**(a)** The positive pLenti-miR-34a A549 cells were shown by imaging with inverted fluorescence microscope.

**(b)** Upregulation of miR-34a in pLenti-miR-34a A549 cells. ***P*<0.01.
