# Supplementary material for: MicroRNA-34a/EGFR axis plays pivotal roles in lung tumorigenesis
Source: Oncogenesis. 2017 Aug 21;6(8):e372–. doi: 10.1038/oncsis.2017.50 (PMC5608916; doi:10.1038/oncsis.2017.50)
Supplement: Supplementary Table S1 [file oncsis201750x7.docx]

Supplementary Table S1 The clinical-pathological features of 60 cases NSCLC patients

| NO. | Gender | Age | Specimen Type | Histologic Type | Lymphatic Invasion | pTNM |
| --- | --- | --- | --- | --- | --- | --- |
| 1 | M | 47 | Pneumonectomy | Adenocarcinoma |  | T1bN0M0 |
| 2 | M | 78 | Lobectomy | Adenocarcinoma |  | T2aN0M0 |
| 3 | F | 67 | Pneumonectomy | Adenocarcinoma |  | T2N1M0 |
| 4 | M | 54 | Lobectomy | Adenocarcinoma |  | T4N0M0 |
| 5 | M | 49 | Lobectomy | Adenocarcinoma | Absent | T2bN2M0 |
| 6 | F | 66 | Pneumonectomy | Adenocarcinoma | Present | T2N2M0 |
| 7 | M | 67 | Lobectomy | Adenocarcinoma |  | T2aN0M0 |
| 8 | F | 62 | Lobectomy | Adenocarcinoma |  | T1aN0M0 |
| 9 | F | 75 | Lobectomy | Adenocarcinoma | Absent | T2aN0M0 |
| 10 | F | 58 | Lobectomy | Adenocarcinoma |  | T2aN1M0 |
| 11 | F | 65 | Lobectomy | Adenocarcinoma | Present | T2aN0M0 |
| 12 | M | 72 | Lobectomy | Adenocarcinoma |  | T2aN0M0 |
| 13 | M | 64 | Lobectomy | Adenocarcinoma | Absent | T1aN0M0 |
| 14 | F | 62 | Lobectomy | Adenocarcinoma | Present | T2aN0M0 |
| 15 | M | 65 | Lobectomy | Adenocarcinoma |  | T4N2M0 |
| 16 | F | 55 | Lobectomy | Adenocarcinoma |  | T2aN1M0 |
| 17 | M | 50 | Lobectomy | Adenocarcinoma |  | T2aN0M0 |
| 18 | F | 67 | Pneumonectomy | Adenocarcinoma |  | T2N1M0 |
| 19 | M | 67 | Lobectomy | Adenocarcinoma | Absent | T2aN0M0 |
| 20 | M | 71 | Lobectomy | Adenocarcinoma | Present | T1bN0M0 |
| 21 | F | 64 | Lobectomy | Adenocarcinoma | Absent | T2aN0M0 |
| 22 | M | 62 | Lobectomy | Adenocarcinoma | Absent | T2aN0M0 |
| 23 | M | 58 | Lobectomy | Adenocarcinoma | Present | T2aN0M0 |
| 24 | M | 70 | Lobectomy | Adenocarcinoma | Present | T3N0M0 |
| 25 | M | 59 | Lobectomy | Adenocarcinoma | Present | T2aN0M1a |
| 26 | F | 72 | Lobectomy | Adenocarcinoma | Absent | T2aN0M0 |
| 27 | M | 51 | Lobectomy | Adenocarcinoma | Absent | T1bN0M0 |
| 28 | M | 51 | Lobectomy | Adenocarcinoma | Absent | T1aN0M0 |
| 29 | F | 55 | Lobectomy | Adenocarcinoma | Absent | T1bN0M0 |
| 30 | F | 53 | Lobectomy | Squamous cell |  | T3N0M0 |
|  |  |  |  | carcinoma |  |  |
| 31 | M | 54 | Lobectomy | Squamous cell |  | T4N2M0 |
|  |  |  |  | carcinoma |  |  |
| 32 | M | 58 | Lobectomy | Squamous cell |  | T3N2M0 |
|  |  |  |  | carcinoma |  |  |
| 33 | M | 65 | Lobectomy | Squamous cell | Absent | T1bN0M0 |
|  |  |  |  | carcinoma |  |  |
| 34 | M | 65 | Lobectomy | Squamous cell |  | T2aN2M0 |
|  |  |  |  | carcinoma |  |  |
| 35 | M | 67 | Lobectomy | Squamous cell | Absent | T2aN1M0 |
|  |  |  |  | carcinoma |  |  |

Table 3 The clinical-pathological features of 60 cases NSCLC patients

| NO. | Gender | Age | Specimen Type | Histologic Type | Lymphatic Invasion | pTNM |
| --- | --- | --- | --- | --- | --- | --- |
| 36 | M | 67 | Lobectomy | Squamous cell |  | T2aN1M0 |
|  |  |  |  | carcinoma |  |  |
| 37 | M | 69 | Lobectomy | Squamous cell |  | T2aN0M0 |
|  |  |  |  | carcinoma |  |  |
| 38 | M | 71 | Lobectomy | Squamous cell |  | T2aN2M0 |
|  |  |  |  | carcinoma |  |  |
| 39 | M | 61 | Lobectomy | Squamous cell | Present | T1bN0M0 |
|  |  |  |  | carcinoma |  |  |
| 40 | M | 67 | Lobectomy | Adenocarcinoma | Absent | T2aN0M0 |
| 41 | F | 64 |  | Adenocarcinoma | Absent | T2aN2M0 |
| 42 | M | 60 | Lobectomy | Adenocarcinoma |  | T1bN0M0 |
| 43 | F | 57 | Lobectomy | Adenocarcinoma | Absent | T1bN0M0 |
| 44 | M | 65 | Lobectomy | Combined Small Cell | Present | T2bN2M0 |
|  |  |  |  | carcinoma |  |  |
| 45 | M | 77 | Lobectomy | Squamous cell | Present | T2aN0M0 |
|  |  |  |  | carcinoma |  |  |
| 46 | M | 71 | Lobectomy | Adenocarcinoma | Present | T1bN0M0 |
| 47 | F | 64 | Lobectomy | Adenocarcinoma | Absent | T2aN0M0 |
| 48 | F | 55 | Lobectomy | Adenocarcinoma | Absent | T1bN2M0 |
| 49 | M | 61 | Lobectomy | Squamous cell | Present | T1bN0M0 |
|  |  |  |  | carcinoma |  |  |
| 50 | M | 62 | Lobectomy | Adenocarcinoma | Absent | T2aN0M0 |
| 51 | M | 58 | Lobectomy | Adenocarcinoma | Absent | T2aN0M0 |
| 52 | F | 60 | Lobectomy | Adenocarcinoma | Absent | T1bN0M0 |
| 53 | F | 61 | Lobectomy | Adenocarcinoma | Present | T2aN1M0 |
| 54 | F | 67 | Lobectomy | Adenocarcinoma | Present | T2aN1M0 |
| 55 | F | 57 | Lobectomy | Adenocarcinoma | Present | T4N0M0 |
| 56 | F | 54 | Lobectomy | Adenocarcinoma | Absent | T2aN3M0 |
| 57 | F | 66 | Lobectomy | Adenocarcinoma | Present | T1bN0M0 |
| 58 | M | 64 | Lobectomy | Adenocarcinoma |  | T2bN2M0 |
| 59 | M | 49 | Lobectomy | Adenocarcinoma | Present | T1bN0M0 |
| 60 | M | 65 | Lobectomy | Adenocarcinoma | Present | T1bN0M0 |
