# Supplementary material for: MicroRNA-34a/EGFR axis plays pivotal roles in lung tumorigenesis
Source: Oncogenesis. 2017 Aug 21;6(8):e372–. doi: 10.1038/oncsis.2017.50 (PMC5608916; doi:10.1038/oncsis.2017.50)
Supplement: Supplementary Table S2 [file oncsis201750x8.docx]

**Supplementary Table S2: Sequences of siRNAs for EGFR**

| **Name** | **Target mRNA** | **Sequence (5′–3′)** |
| --- | --- | --- |
| siEGFR (-1) | EGFR | GGCTGGTTATGTCCTCATT |
| siEGFR (-2) | EGFR | CCTTAGCAGTCTTATCTAA |
| siEGFR (-3) | EGFR | GTAATTATGTGGTGACAGA |
