# Supplementary material for: MicroRNA-34a/EGFR axis plays pivotal roles in lung tumorigenesis
Source: Oncogenesis. 2017 Aug 21;6(8):e372–. doi: 10.1038/oncsis.2017.50 (PMC5608916; doi:10.1038/oncsis.2017.50)
Supplement: Supplementary Table S3 [file oncsis201750x9.docx]

**Supplementary Table S3: Primer sequences used for amplification**

| **Name** | **Usage** | **Sequence (5′–3′)** |
| --- | --- | --- |
| 18S RNA | qPCR forward | AGGAATTCCCAGTAAGTGCG |
|  | qPCR reverse | GCCTCACTAAACCATCCAA |
| U6 snRNA | qPCR forward | CTCGCTTCGGCAGCACA |
|  | qPCR reverse | AACGCTTCACGAATTTGCGT |
| pri-miR-34a | qPCR forward | CCGCTCGAGGGCTGGTCTTGAACTCCT |
|  | qPCR reverse | CCGGAATTCCACTGGCTACTATTCTCCCTA |
| EGFR | qPCR forward | AGGGTGAGCCAAGGGAGTTT |
|  | qPCR reverse | CGTCAATGTAGTGGGCACAC |
| EGFR-3′-UTR | qPCR forward | CGCATTAGCTCTTAGACCCA |
|  | qPCR reverse | AGTAAGTACCGTGGGGACAT |
| EGFR-3′-mUTR | qPCR forward | GTCCATGTATATTGTCCCTTTGAGCAGAAATTTATCT |
|  | qPCR reverse | GGACAATATACATGGACGAAGCGTTTCTGTAAATGCT |
