# Supplementary material for: MicroRNA-34a/EGFR axis plays pivotal roles in lung tumorigenesis
Source: Oncogenesis. 2017 Aug 21;6(8):e372–. doi: 10.1038/oncsis.2017.50 (PMC5608916; doi:10.1038/oncsis.2017.50)
Supplement: Supplementary Table S4 [file oncsis201750x10.docx]

**Supplementary** **Table S4 The verified and predicted targets of miR-34a**

| **miR-34a Targets** | **Carcinomas** | **Biological effect** |  |
| --- | --- | --- | --- |
| SIRT1 | chronic lymphocytic  leukemia | Increased p53  acetylation and activation |  |
|  |  |  |  |
| BCL2 | hepatocellular carcinoma | Apoptosis |  |
|  |  |  |  |
| SNAIL | epithelial-mesenchymal |  |  |
| c-Kit | lung cance | Inhibition of proliferation and survival |  |
| LMTK3 | breast cancer | Inhibition of proliferation and cell cycle progression |  |
| MYB | immortalised myelogenous leukemia |  |  |
| Notch1 | breast cancer | apoptosis, inhibition of proliferation |  |
| CDK4 | immortalised myelogenous leukemia |  |  |
| CDK6 | immortalised myelogenous leukemia |  |  |
| MEK1 | immortalised myelogenous leukemia | Inhibition of proliferation |  |
| MDM4 |  | Positive regulation of p53 |  |
| E2F3 | acute myelocytic leukemia | Senescence and inhibition of proliferation |  |
| Axl | prostate cancer | Inhibition of proliferation, migration and invasion |  |
| MET | Prostate/ breast cancer | Inhibition of invasion and migration |  |
| Fra-1 | breast cancer | Inhibition of invasion and migration |  |
| PNUTS |  | apoptosis, inhibition of proliferation |  |
| VEGF | colorectal cancer | Inhibition of phosphorylation |  |
| HDAC1 | breast cancer | Apoptosis, inhibition of tumor progression, cell proliferation and cell cycle progression |  |
| HDAC7 | breast cancer | Apoptosis, inhibition of tumor progression, cell proliferation and cell cycle progression |  |
| CD44 | prostate cancer stem cells | Inhibition of differentiation |  |
|  | | | |
